# Factors associated with increased burnout in genetic counseling students

**DOI:** 10.1002/jgc4.70094

**Published:** 2025-08-15

**Authors:** Halle McCormick, Leah Wetherill, Laura Oehlman, Paula Delk

**Affiliations:** ^1^ Department of Medical and Molecular Genetics Indiana University School of Medicine Indianapolis Indiana USA; ^2^ Department of Medical and Molecular Genetics Indiana University Health Physicians Indianapolis Indiana USA

**Keywords:** burnout, cynicism, exhaustion, genetic counseling, self‐efficacy, stress, student

## Abstract

Genetic counseling students face numerous stressors during their graduate program, which can lead to negative outcomes such as burnout. Burnout can negatively impact students' ability to perform well, maintain stamina, and feel competent throughout their training. The Maslach Burnout Inventory (MBI) is a validated survey that assesses three domains that together define burnout: exhaustion, cynicism, and low self‐efficacy. One hundred eighty genetic counseling students from the classes of 2024 and 2025 within the United States and Canada completed a cross‐sectional, quantitative survey which included the MBI validated for students, a list of 19 situations tailored to students in genetic counseling training programs that may cause stress, and three open‐ended questions asking what activities helped reduce stress, including resources provided by the program and resources students would like to be provided. Genetic counseling students endorsed an average of nine situations that caused them “some” or “a lot” of stress. Results revealed that 12% of current students met criteria for having burnout (defined as high exhaustion, high cynicism, and low self‐efficacy); an additional 20% met criteria for two of the three burnout subscales. The most consistent predictors of burnout were the numbers of situations causing a student stress and being in the latter part of training. Thematic analyses revealed that personal activities, social activities, and mental health services helped reduce stress, while support in relation to academics was a desired resource. This study reveals that burnout is experienced by genetic counseling students and is associated with factors such as having a high number of situations causing a student stress or being more than halfway through the graduate program. These results provide insight into areas and methods for genetic counseling graduate programs to attenuate burnout in their students.

**What is known about this topic?**

It is known that graduate students in health care, including genetic counseling graduate students, face many stressors, which can lead to adverse outcomes such as burnout. While burnout has been studied in healthcare students as well as practicing genetic counselors, little is known about burnout in genetic counseling graduate students.

**What this paper adds to the topic?**

This study identifies the presence of burnout in genetic counseling graduate students and factors associated with burnout. It also provides insight into steps genetic counseling programs and students can take to help mitigate burnout.

## 1 INTRODUCTION

Graduate school is intense, with high demands on student time and attention. It is therefore not surprising that graduate students experience high levels of stress compared to the general population (Allen et al., 2021; Bogardus et al., 2022). Students in healthcare graduate programs encounter numerous stressors throughout their studies; along with coursework demands, finances, and adulthood responsibilities, healthcare students must also manage clinical rotations and board exams (Bogardus et al., 2022). This increased workload can lead to multiple adverse effects, including depression, anxiety, and burnout (Allen et al., 2021; Bogardus et al., 2022; Jungbluth et al., 2011).

Early research identified three domains that encompass the dimensions of burnout associated with human service employees: emotional exhaustion, cynicism, and personal ineffectiveness (Maslach & Jackson, 1981). Burnout is now broadly studied in other employee types as well as students pursuing various advanced degrees (Maslach & Leiter, 2016; Schaufeli et al., 2002). The Maslach Burnout Inventory (MBI; Maslach & Jackson, 1981) survey tool to measure burnout has been modified for general use in these broader populations (MBI‐GS; Maslach & Jackson, 1996). The MBI‐GS(S) version was validated to assess burnout in adult college students (Schaufeli et al., 2002) and has been used to study burnout across multiple healthcare graduate programs, including medical, dental, and nursing students (Campos et al., 2012; Dyrbye et al., 2014; Frajerman et al., 2019; Ghods et al., 2023; Kilic et al., 2021; Schaufeli et al., 2002). A review of such studies revealed that burnout poses multiple risks for students, such as poor performance in their program, increased chances of attrition, decreased physical and mental health, and increased suicidal ideation (Maroco et al., 2020).

Genetic counseling graduate students, like other healthcare graduate students, face many stressors, including the demands of scientific coursework, caring for patients in clinics, and managing adulthood (Jungbluth et al., 2011). Yet no studies to date investigate burnout specifically in genetic counseling students, despite the documentation of burnout in the genetic counseling profession. For example, one study reported more than half of genetic counselors experience burnout (Caleshu et al., 2022); in particular, close to half of genetic counselors experience exhaustion (41%) or cynicism (48%), although low self‐efficacy was less common (18%; Johnstone et al., 2016). Another study found burnout to be a significant predictor of compassion fatigue in genetic counselors (Lee et al., 2015). Additionally, genetic counselors experiencing high levels of burnout were found to be almost five times more likely to consider leaving their clinical profession (Bernhardt et al., 2009).

The goal of this study was to use the MBI‐GS(S) to assess burnout in current genetic counseling students. Based on a study of medical students that found perceived stress was the strongest predictor of burnout (Kilic et al., 2021), we also focused on exploring several stress factors potentially associated with increased burnout in genetic counseling students, as knowledge in this is lacking. We hypothesized that the number of stressors experienced by students would positively correlate with their measure of burnout. We also hypothesized that students who reported curriculum‐related factors (clinical rotations, didactic coursework, research/capstone project) as causing the most stress would have higher burnout than students stressed by other factors, given genetic counseling students in a prior study reported academic coursework as the most frequent and second most intense stressor (Jungbluth et al., 2011). Results from this study could guide training programs toward interventions that attenuate burnout in their students. Studies have shown that institutional interventions successfully decreased burnout in healthcare students (Brubaker et al., 2020; Burleson et al., 2023), leading to improved patient care, performance in class, and mental health (Maroco et al., 2020).

## 2 METHODS

### 2.1 Participants

Genetic counseling students from the classes of 2024 and 2025 who were enrolled in a genetic counseling training program in the United States or Canada were eligible to participate. Participants who completed their first year of a 3‐year genetic counseling program (graduating in 2026) or graduated within 1 month of receiving the survey were also eligible. We restricted the sample to current students to most accurately estimate burnout experienced *during* graduate school. Administering the MBI‐GS(S) requires a license distributed by Mind Garden, Inc. A review of recent publications that surveyed genetic counseling students indicated the expected number of responses for this study to be around 200. Therefore, permission to administer 200 surveys was obtained. On April 10, 2024, study information and the survey link were emailed to all genetic counseling program directors within North America via the Genetic Counselor Educators Association (GCEA) listserve. The email invited program directors to forward the study information and survey link to their graduate classes of 2024–2025. A reminder email was sent via the GCEA listserve 2 weeks later. The survey closed on April 24 due to reaching the maximum number of surveys.

### 2.2 Instrumentation

This cross‐sectional, quantitative study was deemed exempt by the Indiana University Institutional Review Board (Protocol #22748). Study data were collected and managed using the Research Electronic Data Capture (REDCap) tool hosted at Indiana University (Harris et al., 2009, 2019). REDCap is a secure, web‐based software platform. A brief description of the study was provided in the email and consent was obtained by participants clicking “Next” on the consent page to continue into the survey. After completing the online, anonymous survey, participants could choose to provide an email address through a separate survey for a chance to be randomly selected to win one of five gift cards. Participants were informed that their email addresses would not be linked to their responses.

The survey consisted of four sections, with the first section assessing respondents' eligibility criteria as described above. The second section assessed burnout using the MBI‐GS(S) (Copyright ©1996, 2016 Wilmar B. Schaufeli, Michael P. Leiter, Christina Maslach & Susan E. Jackson. All rights reserved in all media. Published by Mind Garden, Inc., www.mindgarden.com). The MBI‐GS(S) contains 16 questions on a 7‐point Likert scale (0 = “never,” 1 = “a few times a year or less,” 2 = “once a month or less,” 3 = “a few times a month,” 4 = “once a week,” 5 = “a few times a week,” 6 = “every day”) to quantify the participant's level of exhaustion (5 questions), cynicism (5 questions), and self‐efficacy (6 questions). Example questions include “I feel emotionally drained by my studies,” “I doubt the significance of my studies,” and “in my opinion, I am a good student.” The scoring system is described in Section 2.3.1 below. As suggested by the licensure agreement, “stress and wellness” were substituted for the word “burnout” in the survey title and description to minimize bias to the phrase “burnout.”

**FIGURE 1:**
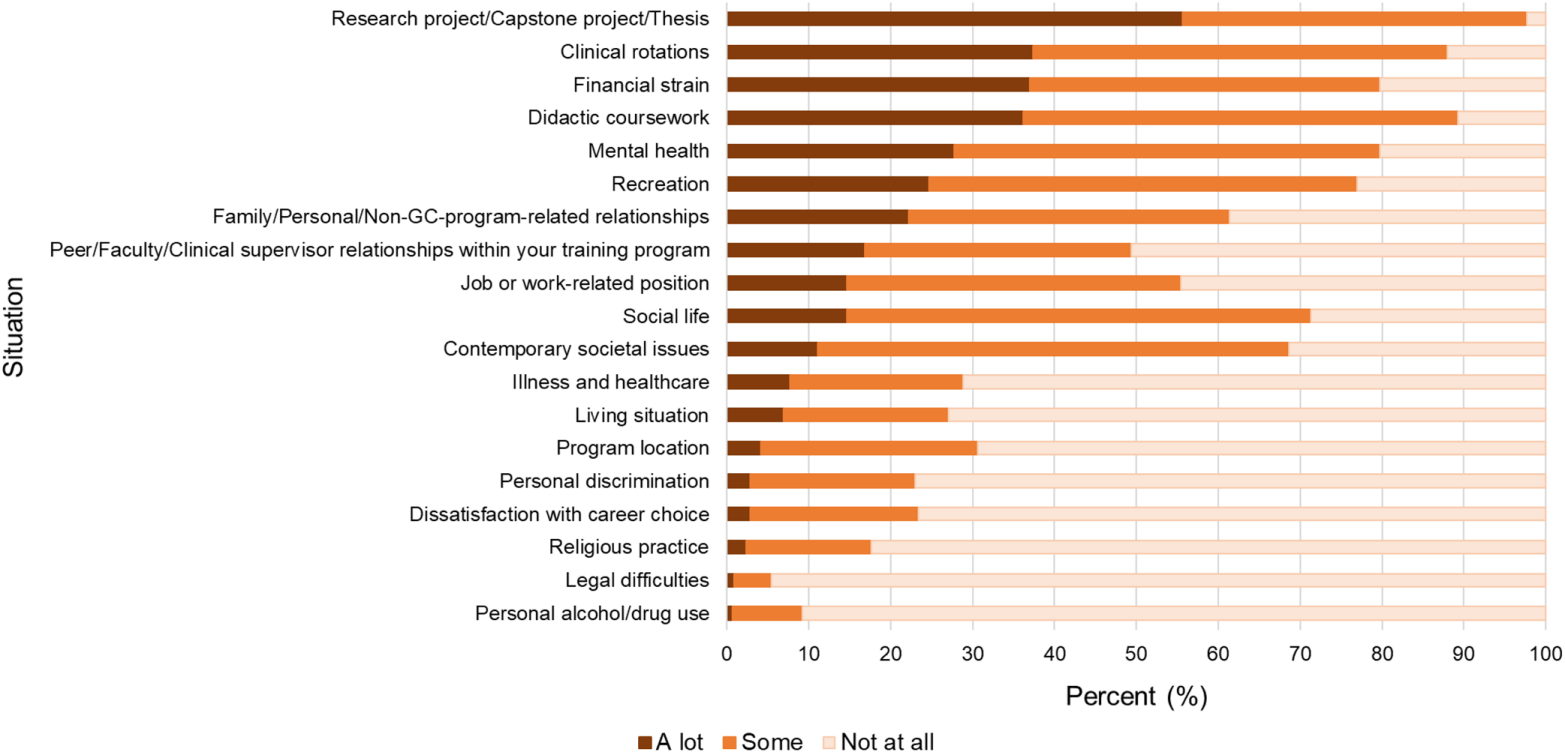
Percent (%) of genetic counseling students who responded that a particular situation was *a lot*, *some*, or *not at all* stressful.

The third section explored situations that might cause stress. Participants were asked how much they currently feel stressed by each of 19 situations (plus “other”) using a 3‐point Likert scale (0 = “not at all,” 1 = “some,” 2 = “a lot,” or N/A if the situation does not apply). Among situations surveyed were academic components (didactic coursework, clinical rotations, research project/capstone/thesis), social components (relationships with family/friends or with peers/faculty/supervisors, social life), extrinsic factors (financial strain, job, living situation, recreation, program location, personal discrimination, legal difficulties, contemporary societal issues), and intrinsic factors (mental health, illness/healthcare, dissatisfaction with career choice, religious practice, personal alcohol/drug use; see Figure 1 for the full list).

The list of 19 situations was modeled and expanded from a previous study on stress and anxiety in genetic counseling students (Jungbluth et al., 2011). Examples were provided for each situation (Appendix S1). Participants then ranked their first, second, and third top stressors. Three open‐ended questions asked participants to list what activities/techniques help them reduce stress, activities/resources currently provided by their program to help students reduce stress, and activities/resources they would like their program to provide to help reduce stress.

The last section collected demographic information (see Appendix S1). Gender was asked according to California State University San Marcos' guidelines on inclusive language in gender identity (https://www.csusm.edu/ipa/surveys/inclusive‐language‐guidelines.html). Race and ethnicity were asked following the National Institute of Health Inclusion Policies (https://orwh.od.nih.gov/our‐work/nih‐inclusion‐policies). One question asked participants to select if they have any of nine disabilities or chronic conditions (e.g., attention deficit, mobility‐related) with the option for “other,” based on the National Center for Women & Information Technology Guide to Demographic Survey Questions (https://wpassets.ncwit.org/wp‐content/uploads/2021/10/29170259/Surveys_DemographicsGuide_10292021.pdf). All demographic factors collected are shown in Table 1. Although the survey was piloted among the investigator's peers prior to data collection, they were not excluded from participating in the survey. All survey questions except the MBI‐GS(S), due to licensure restrictions, are provided in Appendix S1.

**TABLE 1:** Participant demographics.

Demographic variable	Total (*N* = 180)^a^
Time in program	
Less than halfway through	94 (52.2)
Halfway/more than halfway through	86 (47.8)
Graduation timing^b^	
On‐time	166 (92.2)
Taking extra time	6 (3.3)
Region^b^, ^c^	
Midwest	49 (27.2)
Northeast	40 (22.2)
Southeast	24 (13.3)
Pacific	19 (10.6)
Rocky Mtn.	18 (10.0)
Southwest	15 (8.3)
Canadian	12 (6.7)
Age^b^	
21–25	115 (63.9)
26–30	43 (23.9)
31–35	10 (5.6)
36–40	4 (2.2)
41–45	2 (1.1)
46–50	1 (0.6)
Gender^b^	
Woman	161 (89.4)
Man	9 (5.0)
Nonbinary/nonconforming	7 (3.9)
LGBTQIA+^b^	
No	136 (75.6)
Yes	40 (22.2)
Prefer not to respond	1 (0.6)
Race^b^, ^d^	
White	149
Asian	21
Black or African American	4
American Indian or Alaska Native	3
Multiracial^e^	10
Other	5
Prefer not to respond	5
Ethnicity^b^	
Non‐Hispanic or Latino	155
Hispanic or Latino	15
Prefer not to respond	5
At least one disability or chronic condition	
No	104 (57.8)
Yes	76 (42.2)

^a^ Data reported as number (%) unless otherwise stated.

^b^ Data are missing from the following demographics (*n* = number missing): Graduation timing (*n* = 8), Region (*n* = 3), Age (*n* = 5), Gender (*n* = 3), LGBTQIA+ (*n* = 3), Race (*n* = 3), and Ethnicity (*n* = 5).

^c^ States/District/Provinces included in the regions can be found in Appendix S1.

^d^ Participants were able to select more than one option.

^e^ Multiracial is defined as individuals who selected more than one race.

### 2.3 Data analysis

#### 2.3.1 Quantitative data analysis

All analyses were performed using SAS v9.4. Only surveys with complete MBI‐GS(S) responses were included in data analysis. Descriptive statistics were used to report demographic data, including mean, standard deviation (SD), or standard error (SE). Five demographic groups were used in analyses: time in program, LGBTQIA+, gender, race, and disability. Time in program consisted of two groups, depending on whether a person was less than or more than halfway through their program. This was calculated based on the month and year a participant began and ended their program and completed the survey. Recent studies utilizing the MBI‐GS(S) reported higher burnout scores in underrepresented students (Lawrence et al., 2022); we therefore defined demographic groups that are underrepresented in the profession as follows. Participants who identified as LGBTQIA+ were compared to those who did not. Individuals with at least one disability or chronic condition were compared to those with none. To facilitate statistical analysis by gender, due to low participant numbers in all genders aside from “woman,” individuals who chose a response option other than “woman” were combined into one nonwoman group and compared to those who responded “woman.” Participant numbers in all races other than “White” were not high enough to permit statistical analysis between all races. Therefore, individuals who chose a response other than “White” were combined into one non‐White group and compared to those who chose “White.”

The average of the Likert responses for each individual in each of the three domains of the MBI‐GS(S) was utilized, due to the different number of questions in each scale, to create an exhaustion score, cynicism score, and self‐efficacy score (Maslach & Jackson, 1996). Since the self‐efficacy questions are asked in a positive phrase, each self‐efficacy Likert response was reverse‐coded, as advised by Schaufeli et al. (2002). To visually compare the scores between demographic variables of interest and the categorical variable of high vs. low stressors (described below), we standardized each score to have mean = 0 and SD = 1 by subtracting the mean of the particular scale from the participant's value and dividing the difference by the SD of that scale. Due to the primarily unimodal distribution of the three subscales (Appendices [Link], [Link], [Link]) and the large sample size, we utilized a *t*‐test to evaluate differences between categorical variables and each subscale as described below. We also used a *t*‐test to assess differences in each subscale between this sample of genetic counseling students and the sample of practicing clinical genetic counselors from the Johnstone et al. (2016) study.

Following Maslach and Jackson's instructions, individuals with a score above the 66th percentile for exhaustion or cynicism scores were categorized as having high exhaustion or high cynicism, respectively (Maslach & Jackson, 1996). Those below the 33rd percentile for self‐efficacy scores were categorized as having low self‐efficacy (Maslach & Jackson, 1996). Participants scoring high for exhaustion *and* cynicism *and* scoring low for self‐efficacy were classified as having burnout (also called burnout syndrome; Schaufeli et al., 2002).

Situations that participants identified as causing them any (i.e., *some* or *a lot*) stress were summed to determine the total number of stressors for each participant. This quantitative variable was used in analyses described below. To visualize the association between stressors and the three subscales, participants who endorsed more than the mean number of nine stressors (see Section 3.4) were combined into one group (high number of stressors), and those with nine or fewer stressors were categorized as having a low number of stressors. We explored if the three most‐commonly chosen situations ranked as being the most stressful were associated with the three subscales and burnout as follows. The exhaustion, cynicism, and self‐efficacy scores were compared between individuals who chose a situation as being the most stressful factor (yes) and those who did not choose that situation as being the top‐ranked stressor (no) using a *t*‐test. A chi‐squared test was employed to evaluate the association between a particular factor as being the most stressful factor (yes vs. no) and (1) having burnout syndrome (yes vs. no) and (2) each of the five categorical demographic variables.

We estimated the correlation between the total number of stressors and each of the burnout subscales using Pearson's correlation coefficient (*ρ*). We utilized a *t*‐test to explore differences in exhaustion, cynicism, and self‐efficacy scores between the five demographic variables. The five demographic variables and the total number of situations selected as causing *some* or *a lot* of stress were entered into one logistic regression model to predict burnout syndrome (yes vs. no). Only significant (*p* < 0.05) variables were retained, and the model was re‐run using these variables. The odds ratio (OR), upper and lower bounds of the 95% confidence intervals (CI = upper, lower), are reported for the final model. The OR and CI for the odds for an increase of one situation causing stress are reported, as well as for endorsing nine situations, which was the average number in this sample (see Section 3.4). Due to the exploratory nature of this study, we did not correct for multiple testing and utilized an alpha of 0.05.

#### 2.3.2 Qualitative data analysis

Three open‐ended questions were individually scored by the PI (PD) and Co‐PI (HM) using the conventional content analysis method (Hsieh & Shannon, 2005). Scoring systems were developed independently and then discussed by the two authors to create a common coding system to categorize related topics into themes. For example, responses related to connecting with others (talking, messaging friends or family, social activities or time spent with others, cohort discussions, etc.) were coded as a “social activity” theme. In total, six themes were identified for responses to what activities help to reduce stress, nine themes were identified for what resources are provided by programs, and 10 themes were identified for what respondents wished programs would offer (see Table S1 for the full list of themes). When possible, theme titles were kept as similar as possible across the three questions. Data were then individually recoded by the PI and Co‐PI using the finalized coding system. The two authors then compared codes for each response; any discrepancies were resolved between the two authors. Qualitative analysis was for exploratory purposes only, and no statistical analyses were performed.

## 3 RESULTS

The survey reached an estimated 1138 students based on the reported number of students that matched with a genetic counseling program in the United States or Canada in the 2022 and 2023 admissions cycles, according to the National Matching Services, Inc. Purchasing rights to administer the MBI‐GS(S) restricted the maximum number of responses to 200, which represents 17% of the genetic counseling student population. The survey was open for 2 weeks, at which time the maximum allowable 200 responses were received, and the survey automatically closed. Of the 200 responses, 20 were excluded due to either not meeting the inclusion criteria (*N* = 1) or only completing the consent page at the beginning of the survey (*N* = 19), resulting in a 90% completion rate. Of the *N* = 180 remaining participants used in analyses, two completed only the MBI‐GS(S) and one completed all portions of the survey except the demographics.

### 3.1 Demographics

Of the 180 student participants, 48% were more than halfway through their graduate program. The average age was 26 years. The majority identified as a woman (89%), White (85%), and non‐Hispanic or Latino (86%). All regions of the United States were represented in this study, and about 7% of participants attended a genetic counseling program in Canada. Demographic information is summarized in Table 1.

### 3.2 MBI‐GS(S) scores

The MBI‐GS(S) assessed burnout using three subscales: levels of exhaustion, cynicism, and self‐efficacy. Results are reported as the average of the summary score, which could range from 0 to 6. The average score that assessed the student's level of exhaustion was 3.5 (SD = 1.3). There were 34% of participants with scores >66th percentile, representing high exhaustion. The average cynicism score was 2.0 (SD = 1.4), and 34% met criteria for high cynicism. The average self‐efficacy score after reverse coding was 1.6 out of 6 (SD = 0.9), and 34% of participants met criteria for low self‐efficacy.

**FIGURE 2:**
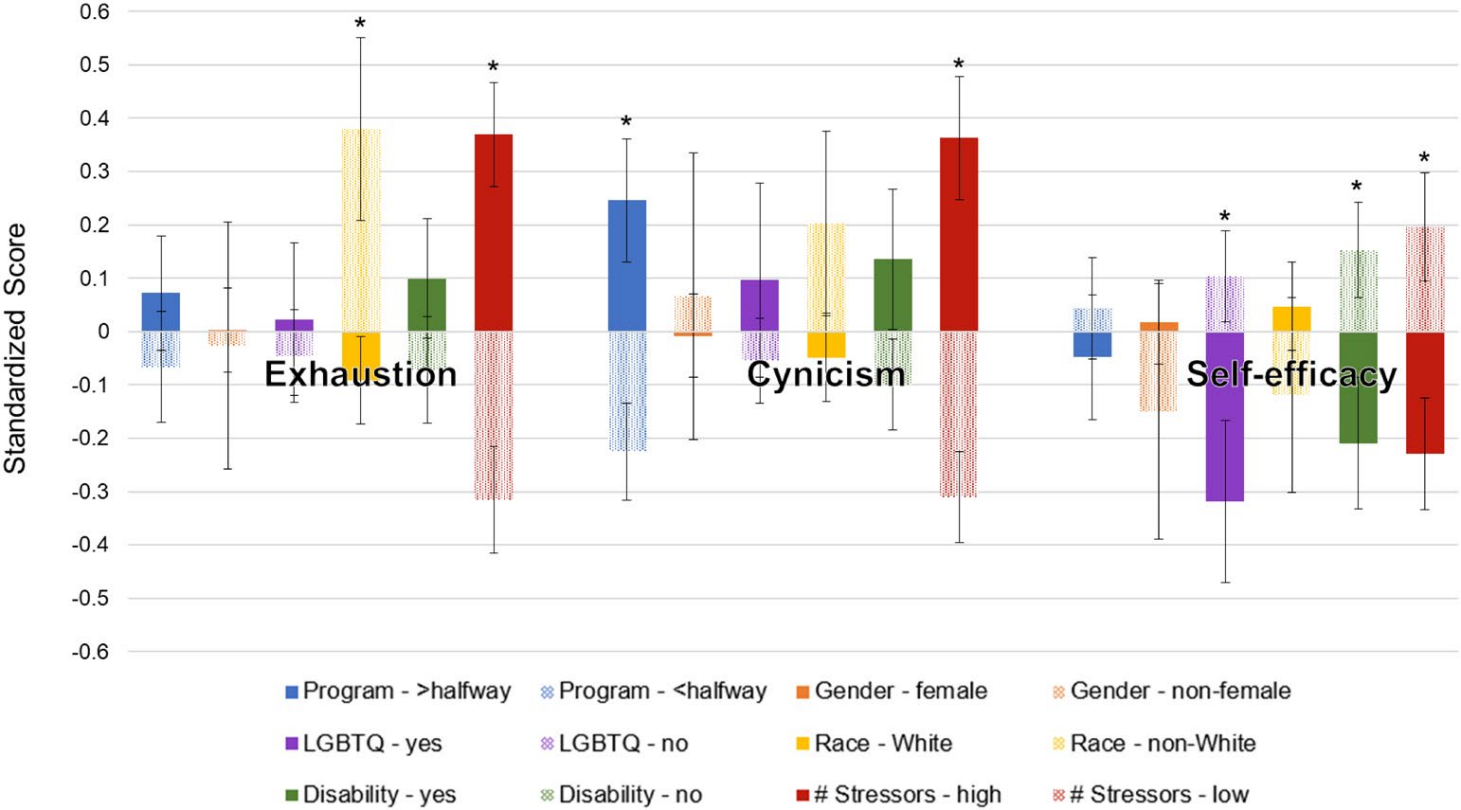
Standardized mean scores of the three MBI‐GS(S) subscales for the five demographic variables along with the categorical number of situations causing a student stress (high ≥9 vs. low ≤9). Asterisks denote significant (*p* < 0.05) *p*‐values.

**FIGURE 3:**
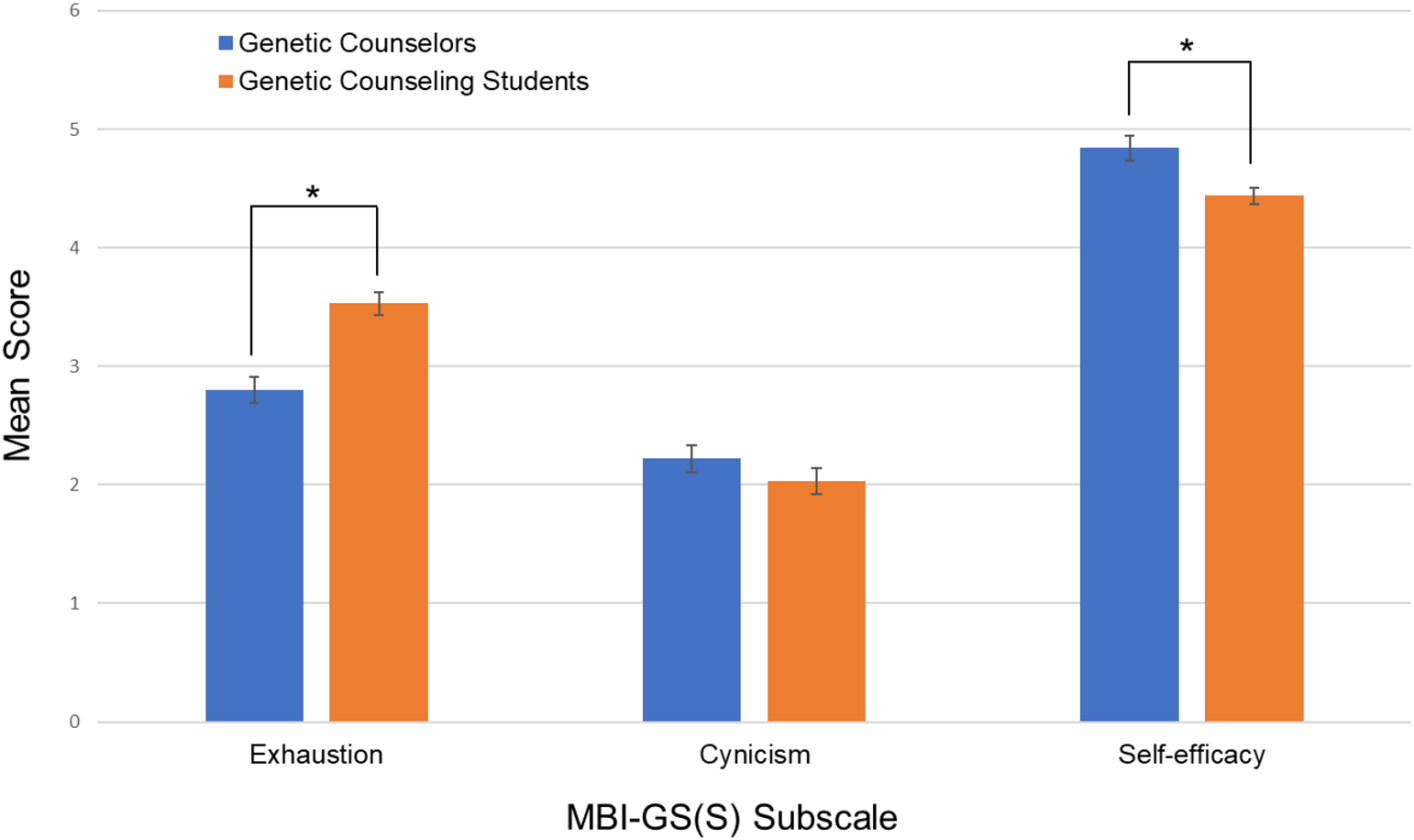
Mean scores of each of the three MBI‐GS(S) subscales for genetic counseling students from the present study compared to practicing clinical genetic counselors. Data for practicing clinical genetic counselors from Johnstone et al. (2016). Asterisks denote significant (*p* < 0.05) *p*‐values.

*T*‐tests were used to assess if the three subscales were different between the five demographic variables. Non‐White individuals had significantly higher exhaustion scores (mean = 4.0, SE = 0.23) than White individuals (mean = 3.4, SE = 0.11, *t*(173) = −2.24, *p* = 0.026). Genetic counseling students more than halfway through their program reported higher cynicism scores (mean = 2.4, SE = 0.17) compared to students less than halfway through their program (mean = 1.7, SE = 0.13, *t*(178) = 3.24, *p* = 0.0014). Those who identified as part of the LGBTQIA+ community or having at least one disability or chronic condition had significantly lower self‐efficacy scores (mean = 4.1, SE = 0.14 and mean = 4.2, SE = 0.11, respectively) compared to those not part of the LGBTQIA+ community or not having a disability or chronic condition (mean = 4.5, SE = 0.08, *t*(174) = −2.39, *p* = 0.018 and mean = 4.6, SE = 0.08, *t*(178) = −2.43, *p* = 0.016, respectively). All other results were nonsignificant (all *p* > 0.12). See Figure 2 for all standardized means for demographic factors and exhaustion, cynicism, and self‐efficacy scores. Current genetic counseling students reported higher exhaustion scores on average compared to practicing genetic counselors from Johnstone et al. (2016) (*t*(1) = 4.85, *p* = 3.03 × 10^−6^) and lower self‐efficacy scores (*t*(1) = −3.17, *p* = 0.0026; Figure 3). There was no difference in cynicism scores (*p* = 0.19).

### 3.3 Burnout prevalence

**FIGURE 4:**
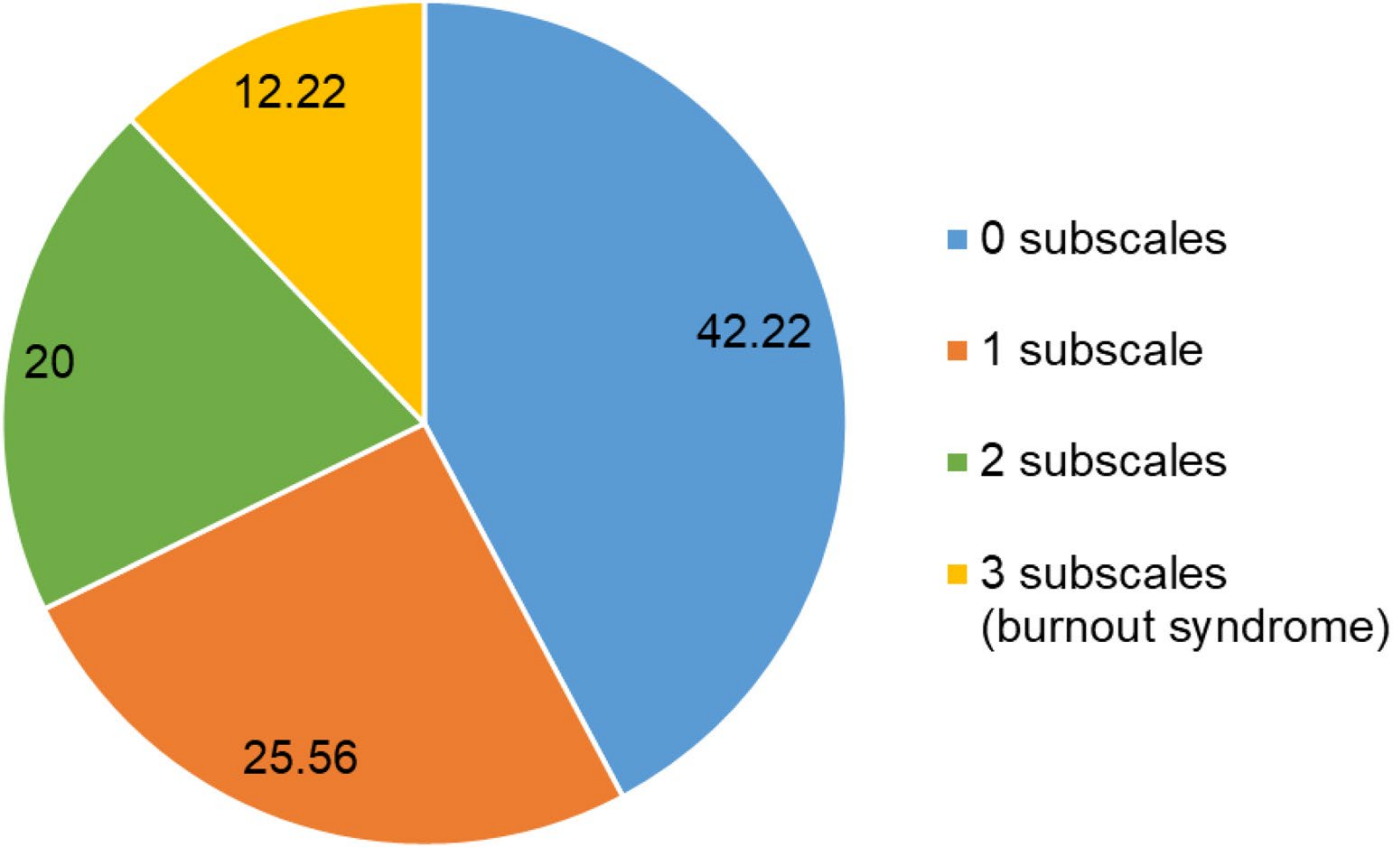
Percent (%) of genetic counseling students meeting criteria for zero, one, two, or all three burnout domains on the MBI‐GS(S).

We found that 26% of current genetic counseling students met criteria for being in the top third (above the 66th percentile) of the sample for exactly one subscale, 20% for two, and 12% met criteria for all three subscales, that is, having burnout (they experienced high levels of exhaustion *and* cynicism *and* low levels of self‐efficacy; Figure 4). We assessed if the five demographic factors and total number of stressful situations predicted burnout syndrome (yes vs. no) in one logistic regression model, and reported results for the final model. Students more than halfway through their program were 2.9 (OR = 2.9) times more likely to have burnout compared to students less than halfway through their program (CI = 1.04, 8.09, *p* = 0.042). Participants with at least one disability or chronic condition were 2.7 times more likely to experience burnout than those without (CI = 1.02, 7.37; *p* = 0.030). For each stressful situation that a student experienced, a student was 1.3 times more likely to have burnout (CI = 1.03, 1.54; *p* = 0.023). Those who experienced the sample average of nine stressful situations were 8.2 times more likely to have burnout (CI = 1.34, 50.20; *p* = 0.008). No other demographic factors predicted burnout syndrome (all *p* > 0.56).

### 3.4 Factors associated with stress and burnout

The most common situation about which students felt stressed was research project/capstone project/thesis (97.7%) followed by didactic coursework (89.3%; Figure 1). Ten of the 19 situations were stressful for at least half of the sample (Figure 1). Of the 19 situations, genetic counseling students endorsed an average of 9.3 (SD = 2.7) situations that caused them any (either *some* or *a lot*) stress. The total number of situations causing stress was positively correlated with cynicism (*ρ* = 0.33), exhaustion (*ρ* = 0.44), and negatively correlated with self‐efficacy (*ρ* = −0.28; all *p* < 0.0002).

We asked participants to rank their top three most‐stressful situations (Appendix S5). Research project/capstone project/thesis was ranked as either the first‐, second‐, or third‐most stressful factor by the greatest proportion of students (68.0%), followed by didactic coursework (49.3%) and clinical rotations (46.9%). The three situations most commonly chosen as the most stressful situation were research project/capstone/project/thesis (31.6%), didactic coursework (21.5%), and clinical rotations (16.9%). Students who chose research project/capstone project/thesis as their most‐stressful situation were more likely to be in the second half of their program rather than the first half (69.6% vs. 30.4%, *χ* ^2^(1) = 15.6, *p* < 0.0001). Conversely, students who chose didactic coursework as their most‐stressful situation were more likely to be in the first half of their program rather than the second half (92.1% vs. 7.9%, *χ* ^2^(1) = 30.7, *p* < 0.0001). Of those who chose clinical rotations as their most‐stressful situation, 26.7% identified as a race other than White versus 73.3% who identified as White (*χ* ^2^(1) = 3.99, *p* = 0.046). There was no association between these three top situations and any other demographic group (all *p* > 0.08).

We tested if exhaustion, cynicism, and self‐efficacy scores were different between students who ranked any of the three top situations as their most‐stressful factor and those who did not. There were no differences in any of the three subscales between students who chose a research‐related situation and those who did not (all *p* > 0.28). Those who indicated didactic coursework as the most stressful had modestly lower self‐efficacy scores (mean = 4.17, SE = 0.16) compared to all other respondents (mean = 4.51, SE = 0.91, *t*(178) = −1.98, *p* = 0.0497). There were no differences in cynicism or exhaustion scales (both *p* > 0.38). Students who chose rotations had higher cynicism scores (mean = 2.51, SE = 0.27) compared to all other respondents (mean = 1.94, SE = 1.42, *t*(178) = 2.00, *p* = 0.048). None of the three most commonly chosen top‐ranked stressful situations were associated with having burnout (all X^2^ *p* > 0.42).

### 3.5 Open‐ended responses

While the primary objective of this study centered around a quantitative analysis, we included three open‐ended questions that asked (1) when you feel stressed, what activities or techniques help you reduce stress, (2) what are some activities or resources your program or university currently provides that are helpful to you in reducing stress, and (3) what are some activities or resources you would like your program or university to provide to help reduce your stress. Responses to these questions are summarized in Appendix S6. Of the 167 responses to the first narrative question, a large proportion (98%) noted personal activities (physical activity, hobbies, and spending time with animals), social activities, and restorative activities (self‐pampering, rest, or therapy/counseling) as techniques they utilize to help them reduce stress; the majority reported utilizing more than one technique. When asked what activities or resources their program currently provides to help students reduce stress, mental health services (36%) and program mentality (35%, e.g., meeting with program staff or mentors, caring leadership, and wellness days or time off) were the two most frequent themes endorsed by the 158 responses. Additionally, 19% of respondents reported they were either uncertain of resources provided by their program or that no resources are provided. When asked what activities or resources they would like their program or university to provide to help them reduce their stress, genetic counseling students suggested adjustments and support within the program (30%) and specifically in relation to academics (23%), burnout or self‐care activities and resources (17%), and social activities (16%), out of the 145 total responses. Some responses regarding academic support mentioned having fewer meetings and “busy work”, providing review sessions for exams, or utilizing a flipped classroom setup. Conversely, 26% of respondents were unsure of what they wished their program or university would provide.

Responses to “other” for factors that may cause stress and the degree of stress they cause were also analyzed. Nine responses pertained to finding a genetic counseling job and the current state of the job market; two related to passing the genetic counseling board exam, and two involved planning for the future, such as a wedding or moving.

## 4 DISCUSSION

To our knowledge, this is the first study to use a validated survey tool to assess burnout in genetic counseling graduate students. Based on these results, more than half of genetic counseling students experience some degree of extreme exhaustion, cynicism, or low self‐efficacy, with 12% of participants meeting criteria for burnout syndrome, according to the MBI‐GS(S). Compared to a 2016 study that used the MBI to quantify burnout in practicing clinical genetic counselors, genetic counseling students in this study reported higher exhaustion scores, lower self‐efficacy scores, and similar cynicism scores (Johnstone et al., 2016). Genetic counseling students may experience different levels of these domains compared to practicing genetic counselors since genetic counseling graduate programs require students to manage coursework, research projects, and clinical rotations, often requiring students to work during nontraditional business hours. Prior studies have also demonstrated that nurses with more years of experience tend to have higher levels of self‐efficacy; therefore, genetic counseling students having lower self‐efficacy compared to experienced genetic counselors is not unexpected (Kallerhult Hermansson et al., 2024; Zarrin et al., 2023). Notably, while 12% of genetic counseling students met criteria for burnout syndrome, an additional 20% met criteria for exactly two of the three burnout subscales. Thus, almost a third of genetic counseling students meet the criteria for having burnout or are close to meeting the criteria. Given that genetic counseling programs in North America currently have an average cohort size of 10 students, these results suggest that, on average, three students in each training cohort are at risk of poor academic performance, increased chance of dropping out of their program, decreased physical and mental health, and increased suicidal ideation (Maroco et al., 2020). Burnout has also been linked with compassion fatigue and contemplation of leaving one's profession (Bernhardt et al., 2009; Lee et al., 2015), with one study demonstrating that nurses who experienced increased burnout early in their profession had more instances of poor cognitive performance, depression, and insomnia 10 years later (Rudman et al., 2020). Additionally, burnout may increase the risk of leaving the genetic counseling profession (de Vries et al., 2023), which may be exacerbated during a poor job market and with recent lower pass rates on the American Board of Genetic Counseling (ABGC) certification exam. Therefore, it is important for genetic counseling programs to strive toward reducing student burnout when possible.

The most consistent and significant predictor for burnout syndrome and all subscales was the number of situations causing genetic counseling students stress. Thus, to effectively decrease burnout in students, decreasing *how many* stressors students experience is more relevant than trying to relieve any one particular stressor. If programs implement practices to reduce the number of situations causing students *some* or *a lot* of stress, the result could be a decrease in student burnout. For example, a study on graduate students in health sciences programs found that utilizing the Pitt Personal Wellness Program significantly decreased the number of stressors experienced by students (Dietz et al., 2023). Offering financial education seminars has also been shown to decrease financial stress in graduate students (Short, 2020). Genetic counseling programs could consider using a multifaceted approach that combines numerous techniques to reduce stress in various aspects of student life, leading to an overall decrease in the number of stressors affecting students.

Although there was not one particular subscale that all underrepresented demographic groups reported less favorable scores for, at least one underrepresented group had significantly lower exhaustion or self‐efficacy scores. These results are similar to medical students who identified as being from an underrepresented ethnic group and reported higher exhaustion and burnout (O'Marr et al., 2022), or who identified as being LGBTQ+ and experienced higher burnout scores (Ryus et al., 2022). Other studies of students in the health profession suggest providing more support from staff and faculty and peer‐based social support to alleviate burnout in students (Clark et al., 2023; Williams‐York et al., 2024).

Prior studies in student populations have explored possible burnout reduction interventions that genetic counseling programs could consider. A recent review identified six studies that documented effective reduction of burnout in nursing students; recreational music‐making, education on mindfulness and resilience, integral‐caring‐holistic‐science curriculum, progressive muscle relaxation, and Acceptance and Commitment Training were effective interventions (Burleson et al., 2023). These interventions are similar to those reported by participants in the present study as useful methods for reducing stress. In another study, students provided with a sunrise alarm clock were observed to have better quality of sleep and decreased burnout scores (Brubaker et al., 2020). Participants from the present study suggested additional areas for program intervention, such as counseling services and financial resources. The interventions utilized in prior studies, along with suggestions from the current participants, are tools that programs could consider implementing.

Students in the second half of their genetic counseling program were more likely to have burnout compared to students in the first half of their training. One possible explanation is that the final year of a genetic counseling graduate program is typically more intense than the first year, leading second‐ or third‐year students to have higher rates of burnout compared to their first‐year peers. Unlike first‐year students, second‐ or third‐year students tend to spend more time in clinical rotations and completing their thesis project (Jungbluth et al., 2011). These activities are primarily done independently rather than as a cohort, with fewer opportunities for empathy and support from cohorts, which could increase stress.

While more advanced students were not more likely to choose rotations as the most stressful factor, they were more likely to rank their research/capstone project as the most stressful factor. Given that roughly 50% of clinical genetic counselors reported being not or minimally interested in research before entering a genetic counseling program (Sikkink et al., 2023), it may not be surprising that research projects would cause students more stress than clinical rotations or didactic coursework. To address this, programs could consider beginning student research projects earlier in training to spread out the workload or offering increased support with research projects near the end of training. As students near graduation, they may also begin searching for jobs and preparing for the ABGC board examination, adding even more stressors to their workload. Providing guidance with acquiring jobs or additional time devoted to applying for jobs might help alleviate stress related to job searching.

### 4.1 Practice implications

Our findings inform genetic counseling graduate programs that genetic counseling students experience burnout. Program leaders should especially be mindful that students more than halfway through their program may be experiencing burnout, and even more so if they are experiencing several stressful situations. Program leaders could consider implementing specific or more frequent interventions that might be helpful for students more than halfway through their programs. For example, periodically asking students to score the 19 situations provided in the present study may help program leaders identify changes in student stress over time and students most at risk for burnout. Multiple participants in our study mentioned access to mental health services, routine check‐ins with program leadership, open‐door policies, meetings with mentors, and wellness days as helpful ways their program aids them in reducing stress. Participants also endorsed wanting their programs to improve support for students, such as providing mentorship and fostering a positive attitude in leadership and clinical supervisors. Other helpful ideas presented by students included the provision of social and self‐care activities along with resources related to stress and burnout. Since a portion of participants were unaware of resources offered by their program or thought no resources were offered, genetic counseling programs could assess the availability and advertisement of such resources to maximize use. These responses offer genetic counseling programs the opportunity to reflect on their current accommodations and utilize these ideas and directions to implement changes.

The Accreditation Council for Genetic Counseling (ACGC) requires that programs have their students complete research and scholarly activities to maintain program accreditation (Accreditation Council for Genetic Counseling, 2023). Providing adequate support throughout the research process could improve self‐efficacy and burnout in students, as supported by one study that reported all students were required to disseminate their genetic counseling program projects and that support for research‐related activities decreased intimidation of the research process (Sikkink et al., 2023).

### 4.2 Study limitations

While our study had a robust response rate, our findings may not be generalizable to all genetic counseling students. Due to the limitation on the number of surveys we were licensed to distribute, the survey was open for only a brief period. This could indicate a response bias of students feeling burnout who initiated the survey soon after learning about it. Our participants were also predominantly White women. Although this reflects the demographic makeup of the genetic counseling field, the sample lacked diversity and therefore inhibits generalizability to nonmajority genetic counseling students. By combining all racially underrepresented respondents, any existing nuances between these groups were lost. Furthermore, combining all nonwoman respondents for analysis purposes conflates man with nonbinary and gender nonconforming. Although research indicates men in the female‐dominated nursing profession have increased risk for stigma, negative stereotyping, stress, and burnout (Alenezi et al., 2024; Zhang et al., 2022), they are reported to experience increased wages and promotions (Gauci et al., 2023; Woo et al., 2022). Thus, combining men with nonbinary and nongender conforming respondents neglects male privilege that may not be afforded to some other respondents.

Additionally, this was a cross‐sectional study. Thus, the time in the calendar year that participants took the survey may have influenced their burnout scores, since students were likely near the end of their semester and may have been experiencing increased responsibilities, such as completing projects, rotations, finals, and preparing for graduation. Furthermore, given that factors such as variability of the job market and sociopolitical climate may alter stress experienced by students, it is possible that burnout scores may differ if the data were collected at a different time. We utilized the validated MBI‐GS(S); however, the remaining survey components were not validated measures for factors that cause students stress, although the situations were adapted from a published study with a large sample size. Also, while we attempted to minimize bias for students answering the MBI‐GS(S) questions by avoiding the word “burnout” in the survey title and description, we cannot eliminate the possibility of participant bias in their responses, which could skew their burnout scores. Likewise, we tried to minimize variability in the interpretation of survey questions by piloting the study and providing participants with definitions and examples, but there remains the possibility that participants did not understand or interpret questions as intended. Some results with *p*‐values close to the threshold of 0.05 would not have been significant had we corrected for multiple testing. As our goal was to provide as much information as possible for future studies, we hope that larger studies will have improved power to confirm or refute our findings. Finally, we organized qualitative responses into detailed themes, but some responses could have been interpreted differently than the participant intended.

### 4.3 Future directions

Future studies could assess burnout in genetic counseling students at different points throughout their training to better understand how levels of burnout may change throughout their program. This may identify factors contributing to higher burnout in students in the second half of training. Longitudinal studies could assess burnout at specific intervals throughout the first few years after graduation. This could identify trends in burnout from graduate school to the early career stage and provide insight into factors that attenuate or accentuate burnout trends. Qualitative research could explore potential protective factors that decrease the likelihood of burnout in genetic counseling students, especially those in the second half of their training. Furthermore, qualitative research could focus on burnout in underrepresented genetic counseling students of both races and genders who are underrepresented in the field to better explore their experiences. It is also worth exploring how programs can assess and monitor burnout in their students, and whether the MBI‐GS(S) is a practical tool programs could consider utilizing.

## 5 CONCLUSION

It is important for genetic counseling graduate programs to be aware that burnout is present in their students and can negatively affect students' health and performance in the program. It is also important for students to be cognizant of whether they may be experiencing burnout so they can consider seeking burnout reduction techniques. Genetic counseling students more than halfway through their program and students with multiple stressors are especially at risk of experiencing burnout. Our study quantified the level of exhaustion, cynicism, and self‐efficacy present in current students and found that they are more exhausted and have lower self‐efficacy than practicing genetic counselors. This study presents several activities and resources genetic counseling programs could consider implementing to decrease the prevalence of burnout in students, and numerous students expressed wanting these changes to be made. Decreasing burnout in genetic counseling graduate students can improve their performance in class, mental health, and quality of patient care they provide.

## AUTHOR CONTRIBUTIONS

Halle McCormick (MS) is a 2025 graduate of the Genetic Counseling Graduate Program (GCGP) in the Medical and Molecular Genetics Department at Indiana University School of Medicine. Paula Delk (MS) is the GCGP Program Director and an ABGC‐certified genetic counselor. Leah Wetherill (MS, PhD) is an assistant scientist with degrees in statistics and addiction neuroscience. Laura Oehlman (MS) is an ABGC‐certified genetic counselor. Delk, Wetherill, and Oehlman are faculty in the Medical and Molecular Genetics Department and work closely with the genetic counseling graduate students. Halle McCormick, Paula Delk, Leah Wetherill, and Laura Oehlman contributed to the project design and methodology. Halle McCormick and Paula Delk were responsible for recruitment and survey distribution. Halle McCormick was responsible for funding acquisition, data collection, and participant gift card distribution. Halle McCormick, Paula Delk, and Leah Wetherill had full access to all study data and take responsibility for the integrity of the data. Data analysis was completed by Leah Wetherill, and she takes responsibility for the accuracy of data analysis. All authors contributed to the revision process and gave final approval for this version to be published. All authors agree to be accountable for all aspects of the work in ensuring that questions related to the accuracy or integrity of any part of the work are appropriately investigated and resolved.

## FUNDING INFORMATION

Indiana University Genetic Counseling Graduate Program, Indiana University School of Medicine.

## CONFLICT OF INTEREST STATEMENT

Halle McCormick, Paula Delk, Leah Wetherill, and Laura Oehlman declare that they have no conflict of interest to disclose.

## ETHICS STATEMENT

Human studies and informed consent: This study was reviewed and granted exemption by the Indiana University Institutional Review Board. Informed consent was obtained from all individual participants before initiating the survey. All procedures were in accordance with the Helsinki Declaration of 1975, as revised in 2000, or comparable ethical standards.

Animal studies: No nonhuman animal studies were carried out by the authors for this article.

## Supporting information


Appendix S1

Appendix S2

Appendix S3

Appendix S4

Appendix S5

Appendix S6

## Data Availability

The data that support the findings of this study are available at reasonable request to the corresponding author.
