# Supplementary material for: Factors associated with increased burnout in genetic counseling students
Source: J Genet Couns. 2025 Aug 15;34(4):e70094. doi: 10.1002/jgc4.70094 (PMC12357068; doi:10.1002/jgc4.70094)
Supplement: Supplementary file 1 — Appendix S1 [file JGC4-34-0-s002.pdf]

Data Dictionary Codebook

Factors that Increase Burnout in GC Students (PID: 40124)

02/13/2025 9:00pm

#	Variable / Field Name	Field Label *Field Note*	Field Attributes (Field Type, Validation, Choices, Calculations, etc.)
Instrument: **GC Student Stress and Wellness Survey** (gc_student_stress_and_wellness_survey) Enabled as survey			
1	[participant_id]	Participant ID	text (integer)
2	[eligibility]	Section Header: *Inclusion criteria*<br>Are you currently enrolled in a genetic counseling training program or a spring 2024 graduate of a genetic counseling training program?	radio, Required<br>1 Yes<br>0 No<br>Stop actions on 0
3	[mbi_gs_s]	Section Header: *Please read each statement carefully and indicate how often you feel this way about your academic work. If you have never had a particular feeling, choose "Never".*<br>Questions from the MBI-GS(S) have been redacted	radio (Matrix), Required<br>0 Never<br>1 A few times a year or less<br>2 Once a month or less<br>3 A few times a month<br>4 Once a week<br>5 A few times a week<br>6 Every day
4	[copyright]	Copyright ©1996, 2016 Wilmar B. Schaufeli, Michael P. Leiter, Christina Maslach & Susan E. Jackson. All rights reserved in all media. Published by Mind Garden, Inc., www.mindgarden.com	descriptive
5	[selfperc_burn]	Section Header: *Please slide the button below to answer the following question.*<br>In your opinion, how much are you currently experiencing academic burnout?(Academic burnout includes feelings of exhaustion due to schoolwork, inadequacy as a student, and negative attitudes towards schoolwork.)	slider (Min: 0, Max: 100)<br>Slider labels: None, Some, A lot<br>Custom alignment: RH
6	[stress1]	Section Header: *Please indicate how much you currently feel stressed by each of the following. Choose "N/A" if the situation does not apply to you. If the situation applies to you but does not cause you any stress, choose "Not at all".*<br>Didactic coursework (e.g., stress due to lectures, course content, studying, homework assignments, your GPA/academic performance, or lack of resources offered through your training program to help you better understand your coursework)	radio (Matrix), Required<br>0 Not at all<br>1 Some<br>2 A lot<br>999 N/A
7	[stress2]	Clinical rotations (e.g., stress due to case preparation, the number of patients you see, the specialty of your rotation, where your rotation site is located, lack of constructive feedback from your supervisor(s), or your performance in clinic)	radio (Matrix), Required<br>0 Not at all<br>1 Some<br>2 A lot<br>999 N/A

8	[stress3]	Research project/Capstone project/Thesis (e.g., stress due to meeting project deadlines, recruiting participants, your performance with the project, lack of direction/assistance, or lack of interest/motivation)	radio (Matrix), Required<br>0 Not at all<br>1 Some<br>2 A lot<br>999 N/A
9	[stress4]	Financial strain (e.g., stress due to debt, student loans, tuition or other program-associated costs, affording basic life necessities like food or housing, or lack of financial support from family/friends)	radio (Matrix), Required<br>0 Not at all<br>1 Some<br>2 A lot<br>999 N/A
10	[stress5]	Peer/Faculty/Clinical supervisor relationships within your training program (e.g., stress due to worrying about maintaining good relationships with your peers/faculty/supervisors, or personally feeling a lack of respect, value, or inclusion by your peers/faculty/supervisors)	radio (Matrix), Required<br>0 Not at all<br>1 Some<br>2 A lot<br>999 N/A
11	[stress6]	Family/Personal/Non-GC-program-related relationships (e.g., stress due to physical distance from your family/significant other(s), mourning a loss, worrying about the health of a loved one, feeling homesick, tension with relationships, accommodating attending family events, or minimal contact with friends and family)	radio (Matrix), Required<br>0 Not at all<br>1 Some<br>2 A lot<br>999 N/A
12	[stress7]	Living situation (e.g., stress due to feeling unsafe/uneasy in your place of residence, your commute from your place of residence to your training program being long or difficult, or your mode of transportation (car/bus/walking) being unreliable)	radio (Matrix), Required<br>0 Not at all<br>1 Some<br>2 A lot<br>999 N/A
13	[stress8]	Program location (e.g., stress due to your training program being in a state other than the one where you spent the most time growing up, being completely virtual, or being in a city that is drastically different in size, culture, or atmosphere than what you were used to prior to graduate school)	radio (Matrix), Required<br>0 Not at all<br>1 Some<br>2 A lot<br>999 N/A
14	[stress9]	Job or work-related position (e.g., stress due to currently having a job during your training program, the number of hours you work, your workload, the location your work, or meeting work-related deadlines)	radio (Matrix), Required<br>0 Not at all<br>1 Some<br>2 A lot<br>999 N/A
15	[stress10]	Mental health (e.g., stress due to personal struggles with mental health, unmanaged/mismanaged mental health, or coping with your mental health)	radio (Matrix), Required<br>0 Not at all<br>1 Some<br>2 A lot

			999 N/A
16	[stress11]	Social life (e.g., stress due to maintaining a social life or feelings of loneliness, isolation, or lacking of friends outside your program)	radio (Matrix), Required<br>0 Not at all<br>1 Some<br>2 A lot<br>999 N/A
17	[stress12]	Recreation (including physical exercise) (e.g., stress due to lack of time to pursue enjoyable activities, lack of recreational and/or physical activities available near you, or local activities not being affordable or convenient)	radio (Matrix), Required<br>0 Not at all<br>1 Some<br>2 A lot<br>999 N/A
18	[stress13]	Dissatisfaction with career choice (e.g., stress due to regretting your decision to become a genetic counselor or considering an alternative career)	radio (Matrix), Required<br>0 Not at all<br>1 Some<br>2 A lot<br>999 N/A
19	[stress14]	Illness and healthcare (e.g., stress due to personal chronic illness, requiring hospitalization/surgery during your program, or an illness preventing you from participating in your program in any way for longer than a week)	radio (Matrix), Required<br>0 Not at all<br>1 Some<br>2 A lot<br>999 N/A
20	[stress15]	Personal discrimination (e.g., stress due to being in a minority group or experiencing mistreatment or bias against you by others at any point during your training program)	radio (Matrix), Required<br>0 Not at all<br>1 Some<br>2 A lot<br>999 N/A
21	[stress16]	Personal alcohol/drug use (e.g., stress due to personal addiction, increased alcohol/drug use since starting your program, or your alcohol/drug use negatively affecting your performance in your program)	radio (Matrix), Required<br>0 Not at all<br>1 Some<br>2 A lot<br>999 N/A
22	[stress17]	Religious practice (e.g., stress due to making accommodations for personal religious practices and events, or lack of time to practice your religion)	radio (Matrix), Required<br>0 Not at all<br>1 Some<br>2 A lot<br>999 N/A
23	[stress18]	Legal difficulties (e.g., stress due to jury duty or difficulties due to accidents, criminal law, civil and political rights, domestic violence, wills, divorce, citizenship, marriage, traffic violations, adoption,	radio (Matrix), Required<br>0 Not at all<br>1 Some

		intellectual property law, wrongful termination, bankruptcy, or immigration)	2 A lot<br>999 N/A
24	[stress19]	Contemporary societal issues (e.g., stress due to political climates, changes in policies and laws, current national or global events, or structural equity)	radio (Matrix), Required<br>0 Not at all<br>1 Some<br>2 A lot<br>999 N/A
25	[stress20]	Other	radio (Matrix)<br>0 Not at all<br>1 Some<br>2 A lot<br>999 N/A
26	[stress20_other]<br>Show the field ONLY if:<br>[stress20] = '1' or [stress20] = '2'	If "other" please describe.	notes
27	[most_stress1]	Section Header: *Of the factors listed on the previous page, please rank the top three factors that cause you stress. Please only rank a factor once. (For example, do not list the same factor as both the 1st and 2nd most stressful)*<br>What is the #1 most stressful factor for you, currently?	dropdown<br>0 Didactic coursework<br>1 Clinical rotations<br>2 Research project/Capstone project/Thesis<br>3 Financial strain<br>4 Peer/Faculty/Clinical supervisor relationships within your training program<br>5 Family/Personal/Non-GC-program-related relationships<br>6 Living situation<br>7 Program location<br>8 Job or work-related position<br>9 Mental health<br>10 Social life<br>11 Recreation (including physical exercise)<br>12 Dissatisfaction with career choice<br>13 Illness and healthcare<br>14 Personal discrimination<br>15 Personal alcohol/drug use<br>16 Religious practice<br>17 Legal difficulties<br>18 Contemporary societal issues

			19 Other
28	[most_stress2]	What is the 2nd most stressful factor for you, currently?	dropdown<br>0 Didactic coursework<br>1 Clinical rotations<br>2 Research project/Capstone project/Thesis<br>3 Financial strain<br>4 Peer/Faculty/Clinical supervisor relationships within your training program<br>5 Family/Personal/Non-GC-program-related relationships<br>6 Living situation<br>7 Program location<br>8 Job or work-related position<br>9 Mental health<br>10 Social life<br>11 Recreation (including physical exercise)<br>12 Dissatisfaction with career choice<br>13 Illness and healthcare<br>14 Personal discrimination<br>15 Personal alcohol/drug use<br>16 Religious practice<br>17 Legal difficulties<br>18 Contemporary societal issues<br>19 Other
29	[most_stress3]	What is the 3rd most stressful factor for you, currently?	dropdown<br>0 Didactic coursework<br>1 Clinical rotations<br>2 Research project/Capstone project/Thesis<br>3 Financial strain<br>4 Peer/Faculty/Clinical supervisor relationships within your training program<br>5 Family/Personal/Non-GC-program-related relationships<br>6 Living situation<br>7 Program location<br>8 Job or work-related position<br>9 Mental health

			10 Social life<br>11 Recreation (including physical exercise)<br>12 Dissatisfaction with career choice<br>13 Illness and healthcare<br>14 Personal discrimination<br>15 Personal alcohol/drug use<br>16 Religious practice<br>17 Legal difficulties<br>18 Contemporary societal issues<br>19 Other
30	[stressred_self]	When you feel stressed, what activities or techniques help you reduce stress?	notes
31	[stressred_uni1]	What are some activities or resources your program or university currently provides that are helpful to you in reducing stress?	notes
32	[stressred_uni2]	What are some activities or resources you would like your program or university to provide to help reduce your stress?	notes
33	[program_start]	Section Header: *Please respond to the following demographic questions.*<br>In what month and year did you start your genetic counseling training program?	dropdown<br>0 January 2021<br>1 February 2021<br>2 March 2021<br>3 April 2021<br>4 May 2021<br>5 June 2021<br>6 July 2021<br>7 August 2021<br>8 September 2021<br>9 October 2021<br>10 November 2021<br>11 December 2021<br>12 January 2022<br>13 February 2022<br>14 March 2022<br>15 April 2022<br>16 May 2022<br>17 June 2022<br>18 July 2022<br>19 August 2022<br>20 September 2022

			21 October 2022<br>22 November 2022<br>23 December 2022<br>24 January 2023<br>25 February 2023<br>26 March 2023<br>27 April 2023<br>28 May 2023<br>29 June 2023<br>30 July 2023<br>31 August 2023<br>32 September 2023<br>33 October 2023<br>34 November 2023<br>35 December 2023
34	[program_end]	In what month and year did/do you anticipate finishing your genetic counseling training program?	dropdown<br>36 January 2024<br>37 February 2024<br>38 March 2024<br>39 April 2024<br>40 May 2024<br>41 June 2024<br>42 July 2024<br>43 August 2024<br>44 September 2024<br>45 October 2024<br>46 November 2024<br>47 December 2024<br>48 January 2025<br>49 February 2025<br>50 March 2025<br>51 April 2025<br>52 May 2025<br>53 June 2025<br>54 July 2025<br>55 August 2025<br>56 September 2025<br>57 October 2025<br>58 November 2025

			59 December 2025<br>60 January 2026<br>61 February 2026<br>62 March 2026<br>63 April 2026<br>64 May 2026<br>65 June 2026<br>66 July 2026<br>67 August 2026<br>68 September 2026<br>69 October 2026<br>70 November 2026<br>71 December 2026
35	[program_time]	Do you anticipate graduating "on-time" or taking extra time to complete your degree requirements?	radio<br>0 On-time<br>1 Taking extra time
36	[region]	In what region is your genetic counseling training program located?	dropdown<br>0 Northeast (CT, DC, DE, MA, MD, ME, NH, NJ, NY, PA, RI, VT)<br>1 Southeast (AL, AR, FL, GA, KY, LA, MS, NC, SC, TN, VA, WV)<br>2 Midwest (IA, IL, IN, KS, MI, MN, MO, ND, NE, OH, SD, WI)<br>3 Rocky Mtn. (CO, ID, MT, NV, UT, WY)<br>4 Southwest (AZ, NM, OK, TX)<br>5 Pacific (AK, CA, HI, OR, WA)<br>6 Canadian (AB, BC, MB, NB, NL, NS, NT, NU, ON, PE, QC, SK, YT)
37	[age]	What is your current age?	text (integer, Min: 18, Max: 100)
38	[gender]	What is your gender identity?	dropdown<br>0 Woman<br>1 Man<br>2 Transgender woman<br>3 Transgender man<br>4 Non-binary/Non-conforming<br>5 Other<br>999 Prefer not to respond
39	[gender_other]<br>Show the field ONLY if:<br>[gender]="5"	If "other" please describe.	text

<table>
<tr><td>40</td><td>[lgbtq]</td><td>Do you identify as LGBTQIA+?</td><td>radio<table><tr><td>1</td><td>Yes</td></tr><tr><td>0</td><td>No</td></tr><tr><td>999</td><td>Prefer not to respond</td></tr></table></td></tr>
<tr><td>41</td><td>[race]</td><td>What is your race? Please select all that apply.</td><td>checkbox<table><tr><td>0</td><td>race___0</td><td>American Indian or Alaska Native</td></tr><tr><td>1</td><td>race___1</td><td>Asian</td></tr><tr><td>2</td><td>race___2</td><td>Black or African American</td></tr><tr><td>3</td><td>race___3</td><td>Hawaiian or Other Pacific Islander</td></tr><tr><td>4</td><td>race___4</td><td>White</td></tr><tr><td>5</td><td>race___5</td><td>Other</td></tr><tr><td>999</td><td>race___999</td><td>Prefer not to respond</td></tr></table>Field Annotation: @NONEOFTHEABOVE='999'</td></tr>
<tr><td>42</td><td>[race_other]
Show the field ONLY if:
[race(5)] = '1'</td><td>If "other" please describe.</td><td>text</td></tr>
<tr><td>43</td><td>[ethnicity]</td><td>What is your ethnicity?</td><td>radio<table><tr><td>0</td><td>Non-Hispanic or Latino</td></tr><tr><td>1</td><td>Hispanic or Latino</td></tr><tr><td>999</td><td>Prefer not to respond</td></tr></table></td></tr>
<tr><td>44</td><td>[dis]</td><td>Do you have any of the following disabilities or chronic conditions? Select all that apply or select "None".</td><td>checkbox<table><tr><td>0</td><td>dis___0</td><td>None</td></tr><tr><td>1</td><td>dis___1</td><td>Attention deficit</td></tr><tr><td>2</td><td>dis___2</td><td>Autism spectrum disorder</td></tr><tr><td>3</td><td>dis___3</td><td>Blind or visually impaired</td></tr><tr><td>4</td><td>dis___4</td><td>Deaf or hard of hearing</td></tr><tr><td>5</td><td>dis___5</td><td>Health-related disability</td></tr><tr><td>6</td><td>dis___6</td><td>Learning disability</td></tr><tr><td>7</td><td>dis___7</td><td>Mental health condition</td></tr><tr><td>8</td><td>dis___8</td><td>Mobility-related disability</td></tr><tr><td>9</td><td>dis___9</td><td>Speech-related disability</td></tr><tr><td>10</td><td>dis___10</td><td>Other</td></tr><tr><td>999</td><td>dis___999</td><td>Prefer not to respond</td></tr></table></td></tr>
</table>

			Field Annotation: @NONEOFTHEABOVE='0,999'
45	[dis_other]<br>Show the field ONLY if:<br>[dis(10)] = "1"	If "other" please describe.	text
46	[add_info]	Is there anything else you would like to share with us?	notes
47	[gc_student_stress_and_wellness_survey_complete]	Section Header: *Form Status*<br>Complete?	dropdown<br>0 Incomplete<br>1 Unverified<br>2 Complete
