# Supplementary figures and images for: Factors associated with increased burnout in genetic counseling students
Source: J Genet Couns. 2025 Aug 15;34(4):e70094. doi: 10.1002/jgc4.70094 (PMC12357068; doi:10.1002/jgc4.70094)

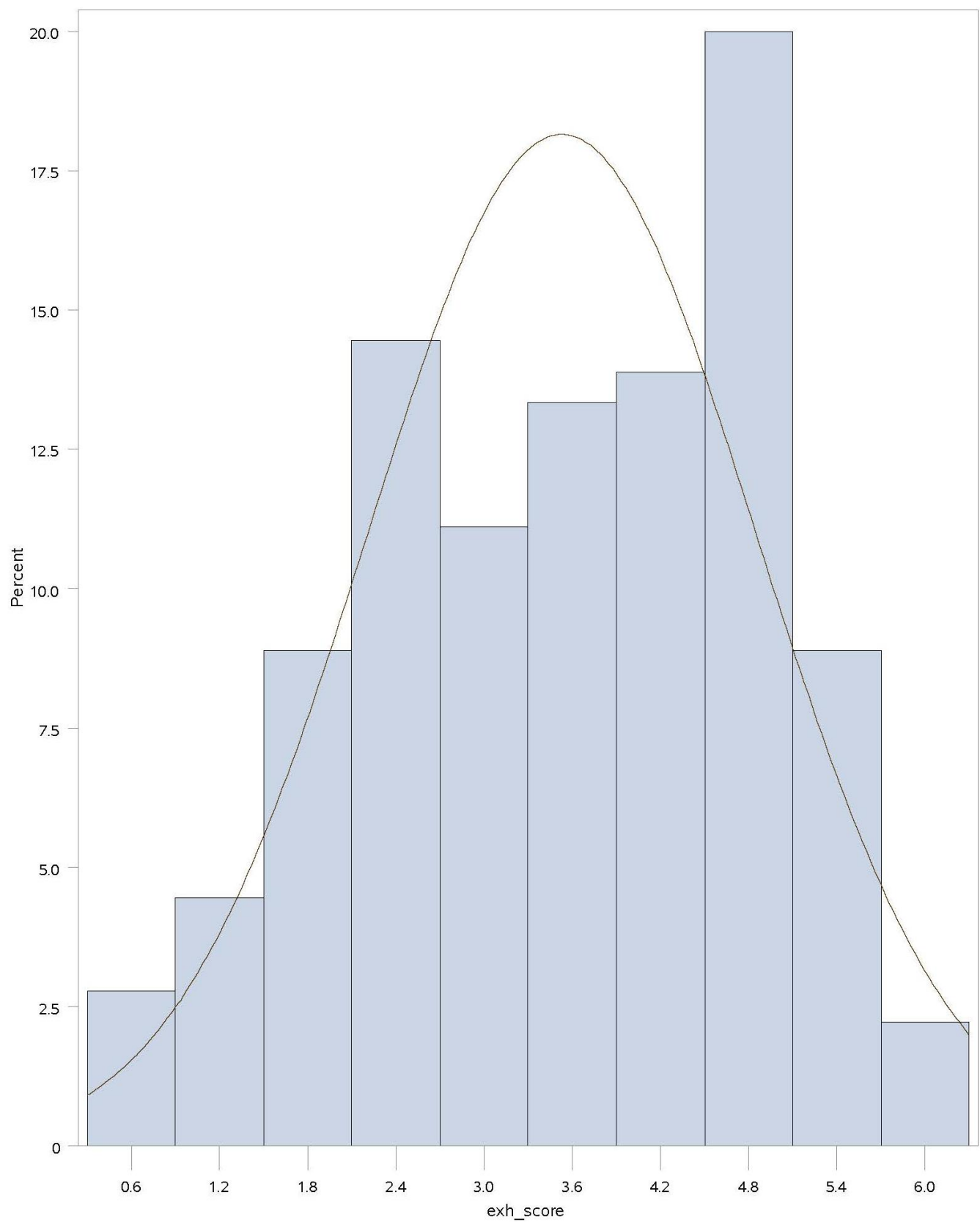

**Figure S2.** Distribution of mean participant exhaustion scores from the MBI-GS(S).

Supplement: Supplementary file 2 — Appendix S2 [file JGC4-34-0-s004.pdf]

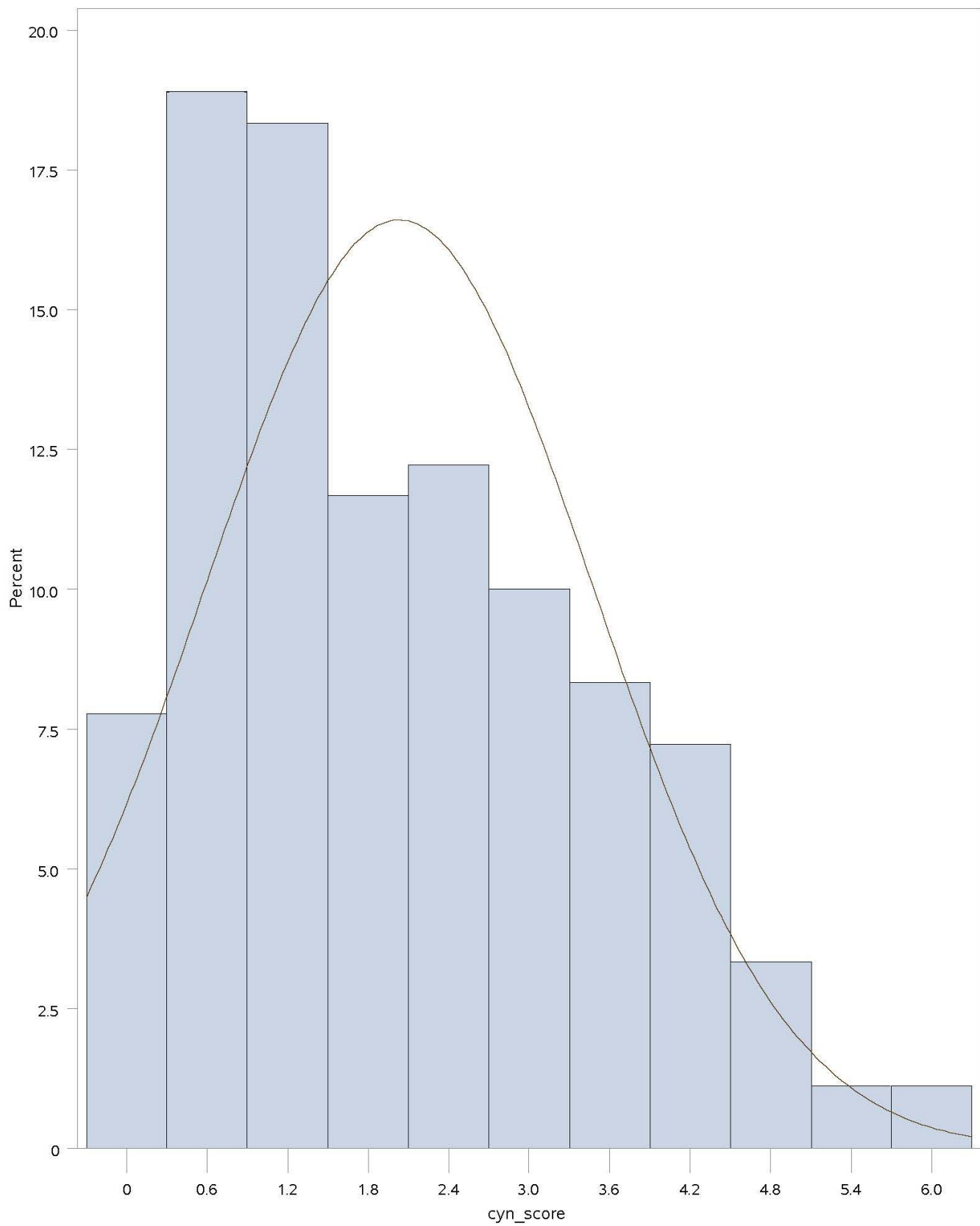

**Figure S3.** Distribution of mean participant cynicism scores from the MBI-GS(S).

Supplement: Supplementary file 3 — Appendix S3 [file JGC4-34-0-s001.pdf]

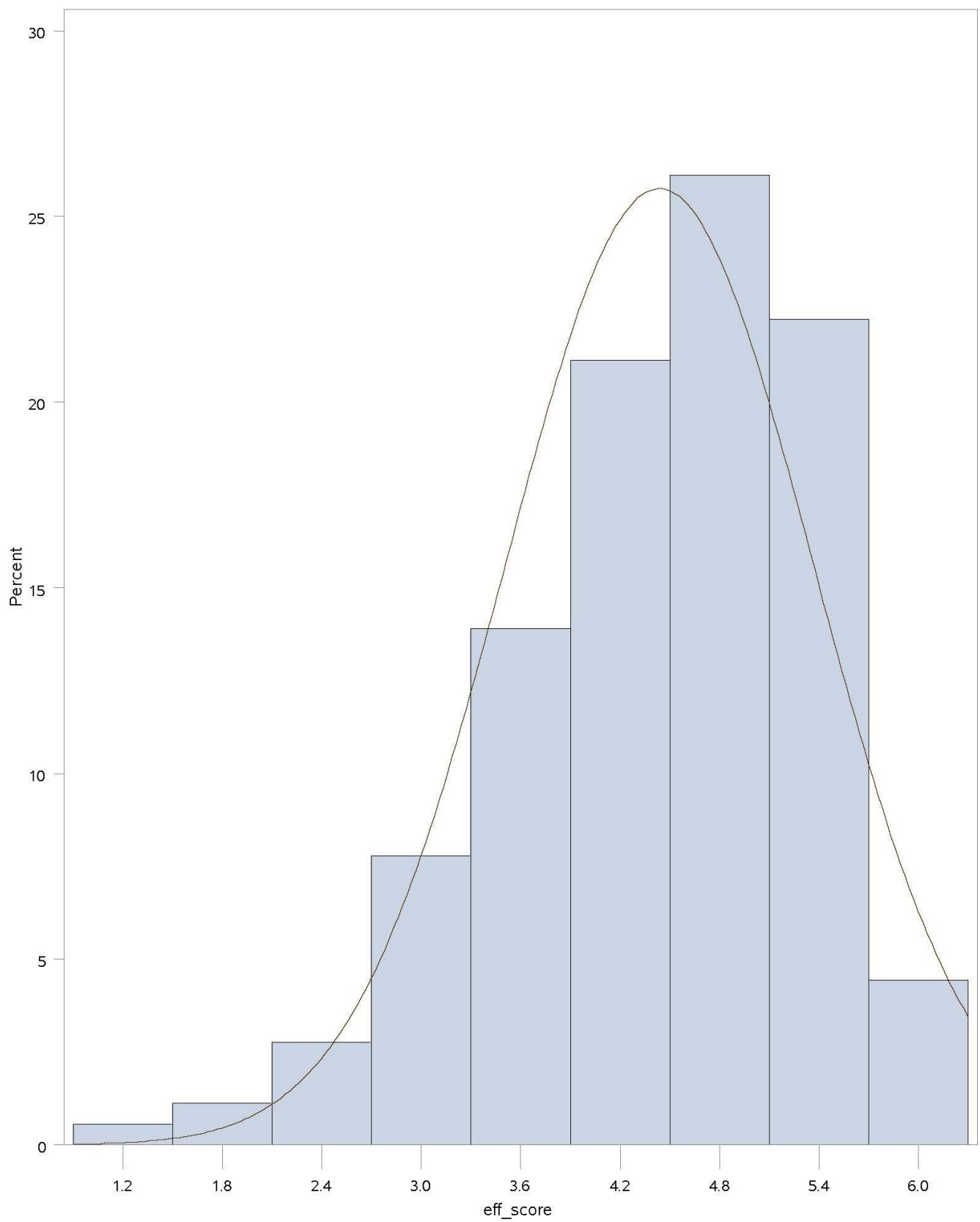

**Figure S4.** Distribution of mean participant self-efficacy scores from the MBI-GS(S).

Supplement: Supplementary file 4 — Appendix S4 [file JGC4-34-0-s005.pdf]
