# Supplementary material for: Factors associated with increased burnout in genetic counseling students
Source: J Genet Couns. 2025 Aug 15;34(4):e70094. doi: 10.1002/jgc4.70094 (PMC12357068; doi:10.1002/jgc4.70094)
Supplement: Supplementary file 5 — Appendix S5 [file JGC4-34-0-s003.docx]

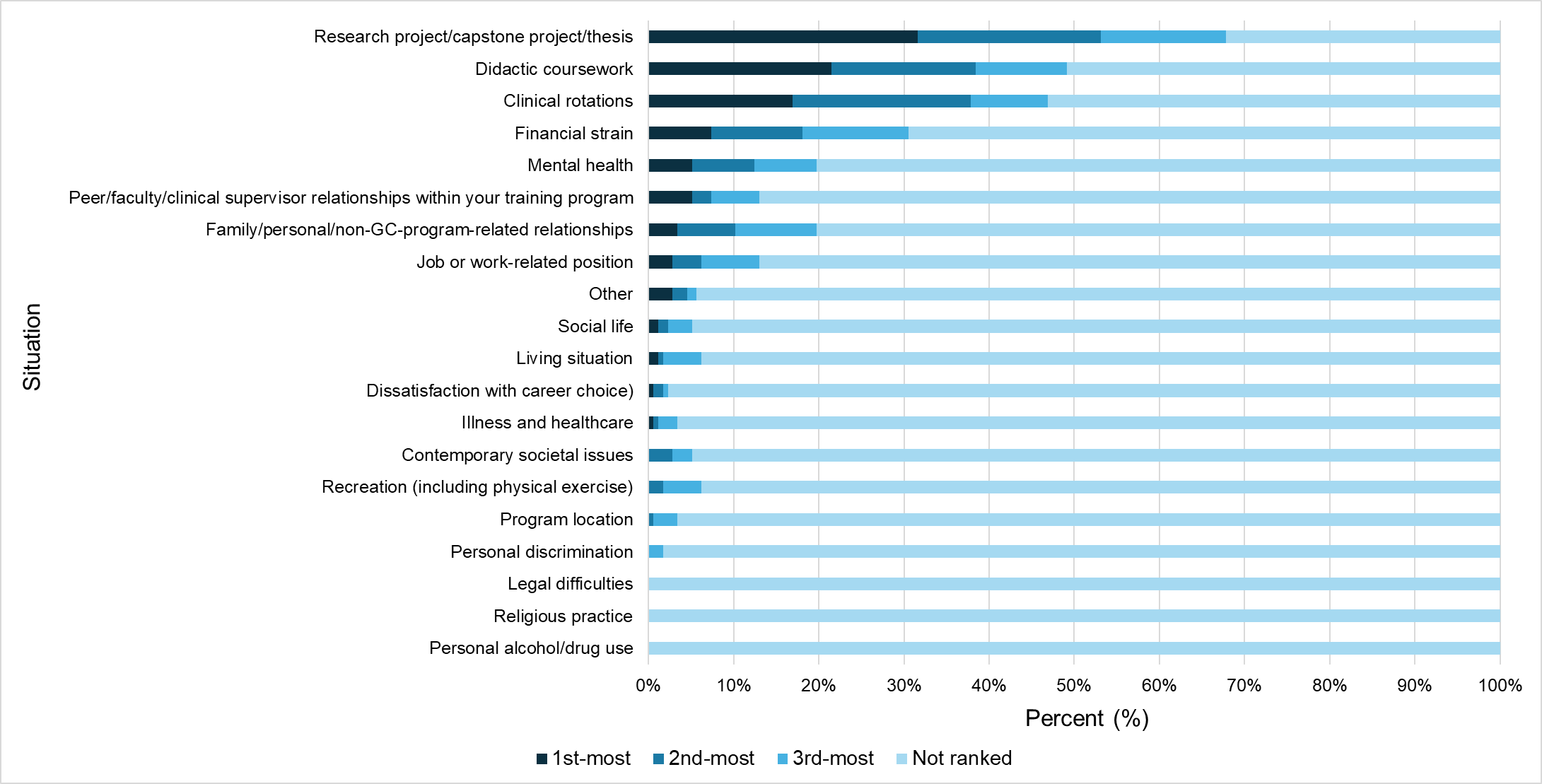


**Figure S5.** Percent (%) of genetic counseling students who ranked a situation as the 1^st^-, 2^nd^-, or 3^rd^-most stressful situation for them, currently.
