# Supplementary material for: Factors associated with increased burnout in genetic counseling students
Source: J Genet Couns. 2025 Aug 15;34(4):e70094. doi: 10.1002/jgc4.70094 (PMC12357068; doi:10.1002/jgc4.70094)
Supplement: Supplementary file 6 — Appendix S6 [file JGC4-34-0-s006.docx]

**Table S1.** Summary of themes identified from three qualitative questions included in the survey.
